# The knowledge and practice of pediatricians in children’s oral health: a scoping review

**DOI:** 10.1186/s12903-020-01198-0

**Published:** 2020-07-25

**Authors:** Virginia Dickson-Swift, Amanda Kenny, Mark Gussy, Colleen McCarthy, Stacey Bracksley-O’Grady

**Affiliations:** 1 Violet Vines Marshman Centre for Rural Health Research, La Trobe Rural Health School, P.O. Box 199, Bendigo, 3552 Australia; 2 College of Social Science, University of Lincoln, Brayford Pool, Lincoln, Lincolnshire LN6 7TS UK; 3 La Trobe Rural Health School, PO Box 199, Bendigo, Vic 3552 Australia

**Keywords:** Oral health, Children, Pediatricians, Dental caries, Dental care, Scoping review, Dental screening

## Abstract

**Background:**

Dental caries is a significant public health problem and one of the most common chronic conditions affecting children. The potential for the non-dental workforce to improve children’s oral health is well documented. For well over a decade, there have been calls for pediatricians to address children’s oral health, but the incorporation of oral health screening, referral, and oral healthcare in pediatric practice remains underdeveloped. Developing action to strengthen the role of pediatricians’ in children’s oral health requires an understanding of their current knowledge and practice. In this scoping review, we aimed to comprehensively map what is known about the knowledge and practice of pediatricians regarding children’s oral health.

**Methods:**

Arksey & O’Malley’s five-stage review process was used to comprehensively map studies undertaken on pediatrician’s knowledge and practice regarding children’s oral health. Key search terms were developed and a total of 42 eligible articles are included in the review.

**Results:**

The studies were conducted in 19 countries. The majority (41/42) were quantitative, with over 90% using self-reported surveys. Only four studies used previously validated survey tools, with most adapting questions from previous studies. Observational designs were used in two studies and one used qualitative methods. Sample size ranged from 15 to 862. Oral health knowledge amongst pediatricians was reported to be mostly poor, with many gaps in key areas including age for first dental visit, dental caries and oral health risk assessments. Studies on the translation of oral health knowledge to practice were limited, with wide variation in rates of assessment. Few studies assessed actual practice.

**Conclusions:**

This scoping review highlights growing international interest in the role of pediatricians in children’s oral health. Findings demonstrate that pediatricians have limited knowledge and understanding in critical areas, including; initial clinical signs of dental caries, recommended age for first dental visit, etiology of dental caries and recommended use of fluorides. Barriers for pediatricians include inadequate education and training, time constraints in practice and lack of referral pathways. Development of a validated tool to assess knowledge and practice is needed. This review provides a starting point to guide future research and areas for systematic reviews.

## Introduction

Internationally, there is a growing focus on the role of the non-dental workforce in improving oral health outcomes, particularly for children [1, 2]. The World Health Organization (WHO) identifies oral health as important for interprofessional practice within the primary health sector [3]. While oral health promotion should be part of the role of a range of health professionals, the key role of pediatricians in children’s oral health has been identified for well over a decade [4, 5]. Recommendations for the role have included screening, anticipatory advice, and referral to dental services before 12 months of age [5, 6]. Despite role identification, routine oral health screening and referral by pediatricians remains limited [7–10].

Dental caries (tooth decay) is one of the most common chronic diseases affecting children. It is a significant public health problem in early childhood, with negative impacts across the lifespan [11–15]. Globally, 60–90% of children are affected, with rates of dental caries higher than childhood asthma [5, 15]. Dental caries is a progressive disease and can be reversed if managed early, but if left untreated, becomes more complex over time [9]. Unmanaged dental caries progresses to cavities with a major impact on child health and wellbeing, including pain, ability to eat and chew, body weight, growth, self-esteem, and communication [14]. The impact of chronic pain/discomfort related to dental caries on child cognitive development has been documented, with poor school attendance and lack of concentration commonly reported [16].

There are multiple determinants for poor oral health in children including social disadvantage, socioeconomic status, age, gender, geographic location, and lifestyle factors [3, 17, 18]. While there has been an overall decline in childhood dental caries rates in developed countries, children in developing countries and children from disadvantaged backgrounds within developed countries continue to experience dental caries at unacceptable levels [18–20].

In 1998, the first national survey on the role of pediatricians in child oral health was conducted in the United States (US) to assess knowledge, attitudes and professional experience [9]. While pediatricians believed oral health could be an important aspect of their practice, few reported any oral health activity. Lack of training was commonly reported. Some have argued that the incorporation of oral health into medical schools and the role of pediatricians, together with appropriate resources, would enhance access to dental care for all children [7, 9]. In 2003, the role of pediatricians in improving the oral health of children was included in a US oral health national call to action [4]. Since that time, the role of pediatricians in oral health has been reinforced and now includes recommendations for pediatric oral health risk assessments from 6 months of age [7–10]. However, there is evidence that the incorporation of oral health in pediatric practice remains underdeveloped [21].

Despite global calls to strengthen oral health in pediatric practice, there have been no reviews that have brought together the evidence on the knowledge and practice of pediatricians regarding children’s oral health. As the first review in this field, our purpose was to complete a broad mapping and synthesis of the evidence base. Scoping review methodology is used to scope a body of literature without excluding studies based on design or quality [22]. This approach aligned with our review aim. Consistent with recommendations of Munn and colleagues [22] our purpose was to identify all available studies, provide commentary on those studies, and identify key knowledge gaps. Authors highlight that scoping reviews are useful as the first step in developing a more focused systematic review and are an important starting point to guide further research [22, 23].

## Methods

The aim of this review was to comprehensively map the literature to answer the following research question “What is known about the knowledge and practice of pediatricians regarding children’s oral health”? Arksey & O’Malley’s [23] five-stage framework guided the review: identifying the research question, identifying relevant studies, study selection, charting and collating the data, and summarising and reporting the data.

An initial search of Google Scholar was carried out to refine the review question and determine key terms. Authors have identified the usefulness of Google Scholar in the initial planning stages for any review [24]. A specialist health librarian assisted with search term development: “knowledge” OR “training OR program” OR “care” OR “practice” OR “education” OR “dental education” AND “oral health” OR “dental care” OR “dental health” OR “dental caries*” OR “oral disease/mouth disease” AND “pediatric*” OR “paediatric*” AND “child*” OR “infant” OR “early childhood”*.* Wild cards (in this case*) were used to capture all terms with the same root word. There are a range of practitioners involved in the care of children, including family physicians and general practitioners, however, our review was specifically focused on pediatricians. The terms paediatrician and pediatrician were used to accommodate for differences in spelling.

Inclusion and exclusion criteria, consistent with our review purpose were developed and are outlined in Table 1.

**Table 1:** Inclusion and exclusion for review

Criterion	Inclusion	Exclusion
Time period	January 2000–April 2020	Studies outside these dates
Language	English	Non-English
Type of article	Original research article published in a peer-reviewed journal	Not original research, not peer-reviewed and/or unpublished
Study focus	Knowledge and/or practice of pediatricians concerning child oral health	No reference to knowledge and/or practice or not undertaken with pediatricians
Geographical area of interest	All countries but reported in English	Nil
Setting	All	Nil

Searches were conducted in Medline, OVID, CINAHL, Proquest, Embase, and AHMED, with database choice guided by an expert librarian. The review process followed the Preferred Reporting Items for Systematic Reviews and Meta-Analysis (PRISMA) [22, 25] framework. The PRISMA checklist is included as a supplementary file. Consistent with Arksey and O’Malley’s [23] framework, data were charted using a data extraction tool for each article that included: author, year, title, journal, location, aims and objectives, number of pediatricians in the sample, key findings and conclusions (see supplementary file). The process of data charting was checked by a minimum of two authors.

Consistent with guidelines for the effective reporting of scoping reviews [26] and the Arksey and O’Malley framework [22], the final stage of the scoping review methodology relates to summarising and reporting the data [22]. For this stage, a thematic mapping approach [22] to summarising the key findings were undertaken which enabled the presentation of a narrative account of the existing literature in relation to the key areas of oral health knowledge and practice of pediatricians and barriers to practice (see supplementary file for full details of the included studies).

## Results

The initial search yielded 3174 studies from all databases. After the deletion of duplicates, 2467 results remained. A total of 2071 articles were excluded as non-empirical studies, (many were general policy guidelines), or articles related to general dentists or pediatric dental specialists.

Of the remaining 396 articles selected for title and abstract review, a random selection of 10 was made to assess interrater reliability [27]. Each team member (*n* = 5) independently reviewed the same 10 articles against inclusion and exclusion criteria. Scorer sheets were completed and Kappa’s co-efficient was calculated using Stata™ data analysis software. The kappa-statistic measure of agreement was 0.6190, indicating substantial agreement [28]. Discussion of these 10 articles occurred between all authors which supported the development of shared understandings. To ensure consistency across the review, a decision was made that all abstracts would be reviewed by the same second reviewer (VDS). This process resulted in strong agreement through the review process. Any conflicts between reviewers were discussed by the full team until agreement was reached.

**Fig. 1:**
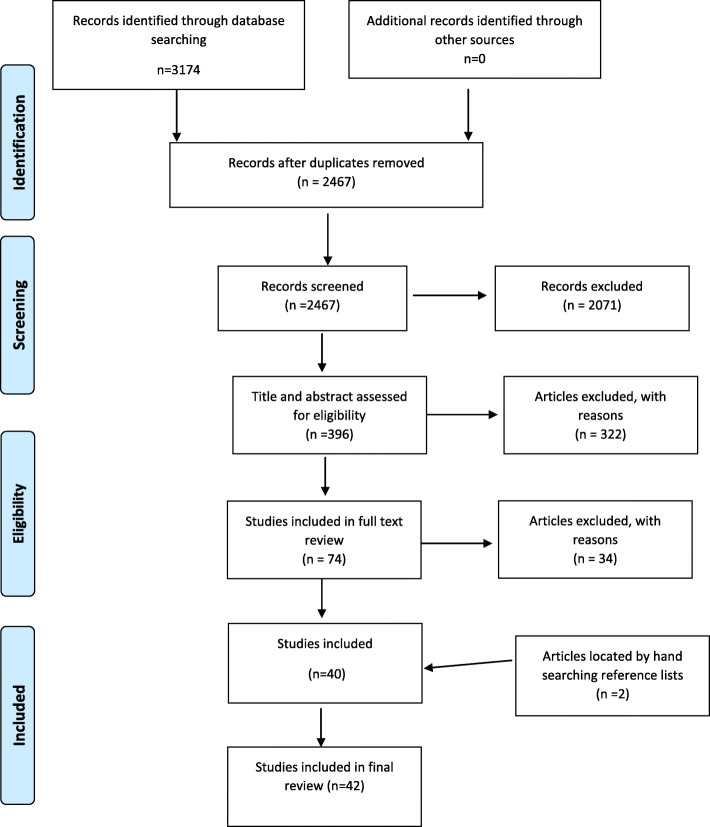
PRISMA flowchart

The 396 articles were retrieved and reviewed by five reviewers (approximately 80 per reviewer). A further 322 articles were excluded as not meeting the criteria. The full text of the 74 remaining articles were evaluated by two researchers (VDS, CM). Another 34 studies were excluded: 19 did not focus exclusively on pediatricians, eight were focused on evaluations of training programs, three were reviews, one was an opinion piece, one included interviews with parents, one examined interprofessional practice, and one was a duplicate. Hand searching of the reference lists of the included studies resulted in another two articles being identified, resulting in a total of 42 articles eligible for inclusion in this review. Figure 1 outlines the review process.

### Key features of included studies

The 42 included studies were mostly undertaken by US and Indian research teams and were published in the latter part of the 2000s: India (11 studies), US (9 studies) Saudi Arabia (3 studies), Brazil, (3 studies), Nigeria (2 studies), and single studies from a range of other countries including UK, Australia, Belgium, Germany, Iran, Singapore, Italy, Taiwan, Montenegro, Lebanon, Trinidad/Tobago, Turkey, Tehran, and one that covered a number of European countries. A total of 39 (92%) used cross-sectional self-reported surveys ranging from 18 to 101 items, via telephone, hard copy, mail and web based. The remaining three studies included two observational studies [29, 30] and one qualitative study [31].

The surveys were often developed by the researchers based on studies conducted in similar countries. For example, the US National Survey developed by Lewis [9] and colleagues was used as a basis for studies in the US [9, 32–34] and Australia [35] whilst tools developed for the Indian context [36–39] were frequently adapted for studies in India and the Middle East. Only four studies reported using validated tools [7–9, 34]. More than half (51%) of the survey studies provided no detail of the tool validation or pre/pilot testing. The survey tools predominately focused on the domains of knowledge and practice. Knowledge included questions related to age for first dental visit, oral health risk assessments, including caries etiology, risk factor prevention, toothbrushing, and fluoride supplementation. Practice questions included oral health screening and examination, anticipatory guidance, referral to dentists and fluoride application.

Two studies used observational designs to assess pediatrician’s ability to identify visible plaque on the teeth of young children [29] and to identify risk for the development of early childhood caries (ECC) [30]. A qualitative interview study conducted by Karasz and colleagues [40] explored the barriers and facilitators to caries prevention for young children of immigrant Bangladeshi families in New York.

Whilst many of the studies published between 2000 and 2010 focused on the US and India, studies published in the past 3 years (2017–2020) have been undertaken in 9 new countries (Australia, Brazil, Lebanon, Taiwan, Europe, Saudi Arabia, Singapore, Trinidad/Tobago and Montenegro).

Sample sizes for the included studies ranged from 15 to 862 pediatricians. The qualitative study undertaken by Karansz and colleagues had the smallest sample (15) appropriate for a study of this type. In the survey-based studies (*n* = 39) half of the sample sizes were relatively small with 19/39 (49%) with samples of 100 or less. A total of 31 % (12/39) had samples between 100 and 300 and 8/39 (20%) studies with samples over 300. The largest sample (*n* = 862) was in the US national survey in 2000 (response rate of 62%) [9]. Most studies did not report on sample sizes relative to the population of pediatricians in each of the areas in which the surveys were carried out.

### Synthesis of study findings

Munn et al. [22] reinforces the need for authors using scoping review methodology to comprehensively describe the key findings from included studies. The contents of the 42 identified articles were thematically analyzed, and clustered into three groupings: knowledge, practice and barriers.

### Oral health knowledge

Oral health knowledge of pediatricians was often reported as inadequate. Authors from the US highlighted a lack of knowledge in relation to developmental oral health [33] and age for first dental visit [34]. Lack of knowledge of dental caries was reported from a researcher developed survey of 75 pediatricians in India, [36] and in Saudi Arabia, significant gaps in knowledge regarding the use of pit and fissure sealants and fluoride supplementation was reported from a researcher developed survey of 363 pediatricians. Similar findings were reported in Nigeria [41], Brazil [42], Tehran [43].

Authors of the reviewed studies indicated limited knowledge and understanding of the transmission of bacteria from mother to child in the etiology of dental caries. In a large European study, 22% of respondents reported being unsure about bacterial transmission [44]. Two Indian studies explored pediatrician knowledge of bacteria transmission. In one study [36], less than half of pediatricians knew that bacteria associated with dental caries could be transmitted from mother to child. In the second [45], half of respondents disagreed that bacteria associated with dental caries are transmitted from mother to child. Poor knowledge of transmission was reported in other studies [46, 47].

Data from a large European study, using a web-based survey with 510 European pediatricians who were members of EAPRASnet (European Pediatric Research In Ambulatory Setting Network), indicated that 25% of respondents were unaware of the initial clinical signs of dental caries (i.e. early or white spot lesions) [44]. From a recent Australian survey adapted from the American Academy of Pediatrics [48] only 17.1% of Australian pediatricians rated their ability to assess dental caries as excellent and only 7.6% felt confident in their ability to assess plaque buildup [35]. A lack of awareness of early lesions was found by Nassif and colleagues [49] in Lebanon, with 25% of pediatrician respondents unaware.

Emmi and colleagues [50] in Brazil used a researcher developed survey of 70 pediatricians with 90% of respondents having outdated knowledge in areas such as fluoride use. In the US, Lewis and colleagues [7] reported that 94% of study respondents were confident in determining the need for fluoride supplementation and providing the right fluoride dose. Whilst in the UK, Kalkani and colleagues [51] found that only one in five pediatric postgraduate trainees could correctly identify the correct dosages for fluoride supplements. In India, a study by Shetty & Dixit [52] found that 92% of pediatricians surveyed knew about fluoride use as a preventative measure, although no data were collected on how many recommended fluoride in their practice. German pediatricians recommended the simultaneous use of fluoride supplements and fluoride toothpaste 45.9% of the time for children in the first 3 years without information about other fluoride sources or water fluoride levels [53].

### Oral health practice

There were only two studies that used observational methods to examine pediatrician’s ability to assess for early childhood caries [29, 30]. Dumas and colleagues [29] assessed pediatricians’ ability to identify visible plaque. These results were then compared to an examination undertaken by a dental hygienist. Pediatricians (*n* = 28) identified visible plaque on 39% of children (*n* = 118), with low levels of agreement with a dental hygienist. In the US, 1288 pediatricians completed an oral health risk assessment and referral tool (POORT) The results showed low referral rates for at risk children.

From a researcher developed survey [52] of 84 pediatricians in India, only 24% of respondents considered themselves knowledgeable on oral health and reported direct impacts on rates of routine oral health examination. Sezer et al. [54] established a relationship between pediatricians’ knowledge and dental referral rates; pediatricians with greater knowledge reporting higher rates of referral.

Studies conducted in the US and India found high levels (~ 80%) of agreement that pediatricians had an important role in identifying dental problems and moderately strong agreement that they should provide counselling on the prevention of dental caries [9, 36, 55, 56]. In a national survey undertaken in the US by Lewis [7], 90% of pediatricians agreed that they should examine children’s teeth for dental caries, but only about half (54%) reported examining the majority of 0–3-year-old children. In countries other than the US, variable rates of examination for oral disease were reported. Rates ranged from 90% in a Canadian study [39] to between 13 and 60% in Indian studies [38, 56, 57]. In an Indian study, [52] researchers identified practice setting differences, with 65% of pediatricians practicing in both teaching institutions and private practice, but only 32% of those working exclusively in private practice reported screening for dental disease. Selective examination for oral diseases was found by Bozogemehr and colleagues [58]; oral exams were conducted by 88% of their respondents but only if the child had a reported problem. In Australia pediatricians reported undertaking general assessments including chest (71%) and cardiac (77%) examinations, but only 40% routinely conducted oral health assessments [35].

Karasz [31] reported that while most pediatricians were aware of the guidelines for referral of children to a dentist before 12 months of age, few ensured referral completion. A lack of awareness and implementation of dental referrals for young children was reported across a number of studies [31, 36, 44, 55, 59–63]. Studies from the US showed wide variations in dental referral rates of one-year-old infants ranging from 8 to 45% [34, 47, 60, 64]. Virdi et al. [56] reported that two-thirds of pediatricians surveyed recommended a dental check-up only when dental problems were reported. Similarly, Indira et al. [62] found that only 11% of pediatricians routinely advised caregivers about the child’s first dental visit before age one.

Prescribing of fluoride supplements and/or application of concentrated fluoride varnish (CFV) was variable across the studies. Authors that included reports on the application of CFV, found rates of use were low. Lewis et al. [7] reported that while 20% of pediatricians agreed that they should apply CFV routinely, only 4% of pediatricians did this regularly. There appears to have been only limited increase in CFV application over the past 6 years, with more recent findings from the US indicating 12% of pediatricians apply fluoride varnish treatment to children between three and six [65]. Reported rates of prescribing fluoride supplements showed large variation from nearly 90% of pediatricians surveyed in one study prescribing fluoride supplements in Italy [66] to only 7% in Belgium [67].

### Barriers that impacted on oral health knowledge and practice

There was a lack of reporting on the key barriers that impacted on oral health and knowledge and practice with most studies focusing on inadequate education and training opportunities for pediatricians [7, 9, 33, 36, 41, 43, 51, 55, 68, 69]. In a Brazilian study, Balaban et al. 83.4% classified the oral health content in their medical education as either non-existent or deficient [68]. Some authors reported that increased training led to improved pediatrician confidence, and knowledge of dental topics, however, little effect on actual practice was reported [66, 70]. Despite this, authors of many of the included studies made recommendations for further training [7, 8, 33, 41, 51, 52, 71, 72].

A major practice barrier was related to time. Lewis [8] found the majority (84%) of respondents in their study provided anticipatory oral health guidance to parents or carers, however, only 39% felt they had adequate time to fully cover all of the guidance they wanted to impart during a visit. In a follow-up study by the same authors, over 90% of pediatricians educated families about preventative oral health [7].

In the US, one of the persistent barriers to referral was the medical/insurance system [7–9]. Parental acceptance of dental advice and low likelihood of caregivers implementing dental referrals or recommendations were also identified [31, 34]. Other barriers identified included costs, long waiting times for dental treatment [64] and lack of dental providers that were willing to see infants and young children.

## Discussion

This is the first review to map and synthesize the evidence on the knowledge and practice of pediatricians regarding children’s oral health. Our purpose was to identify all available studies without excluding studies based on study design or quality. Consistent with scoping review methodology, our purpose was not to provide a detailed meta-synthesis or meta-analysis of study findings, rather, to broadly scope the literature, provide a synthesis of key findings, and recommendations for further research [22]. Scoping reviews are intrinsically different to systematic reviews. The goal of a systematic review is to identify and synthesise studies, with a strong emphasis on quality appraisal. They commonly include meta-analysis, where data from studies with a high level of evidence, such as randomized controlled trials, are pooled to identify common effect [73]. By using a scoping review methodology, we did not exclude studies based on quality, rather provided a broad synthesis of the field as a useful starting point to inform future systematic reviews and other research efforts [22, 23]. Documenting all relevant studies on the knowledge and practice of pediatricians regarding children’s oral health is a strength of this review.

Most of the studies included in this review were cross-sectional and used self-reported surveys to evaluate pediatrician’s knowledge and practice. Only two of the reviewed studies used observational designs. While self-reported surveys of knowledge and practice are useful, self-reports of practice may differ from actual practice. Studies that include observation of actual practice and audits of client records would advance knowledge in this field.

A total of 35 (89%) of the studies used researcher developed surveys to explore oral health knowledge and practice. Sample sizes varied across these studies, and few included power calculations. The development of a well-designed, validated tool to assess pediatrician’s oral health knowledge and practice is an important first step. In some studies, surveys were based on the American Academy of Pediatrics (AAP) guidelines for caries-risk assessment and anticipatory guidance for infants and young children [48]. Using established guidelines in survey development would appear to be logical, however, gaining international consensus on a consistent tool would enable pooling of evidence and within country and cross-country comparisons. Techniques to build consensus could include international surveys, workshops at international conferences, and the use of the Delphi technique [74]. Validation and testing across different countries would be integral. There was a paucity of qualitative or mixed method studies. Incorporating qualitative methods would be a useful addition to the evidence in this field, to add depth to understandings, particularly in the exploration of barriers to incorporating oral health in pediatric practice.

There was agreement across all studies that there is a role for pediatricians in the promotion of oral health. However, review findings suggest major gaps in oral health education and training. A review of pediatric educational programs should be conducted with oral health content and competencies mapped. In this review, key knowledge deficits were identified in the transmission of bacteria from mother to child in the etiology of dental caries, the clinical signs of early (and therefore reversible) dental caries, and the use of specific interventions such as fluoride therapies. Collectively, the included studies indicate a need for greater formal education and training for pediatricians in oral health and effective interventions. There is a large body of literature that indicates that increased knowledge leads to higher levels of confidence but not necessarily to changes in practice [2, 7–9, 36, 37, 44, 50, 60–62, 66, 70, 75, 76]. This may indicate that other structural or setting-based barriers exist but apart from time-constraints none of the authors explored this in any meaningful way. Future studies are needed to provide more a more detailed understanding of the key barriers to translation of oral health knowledge to pediatric practice.

While a number of studies reviewed focused on pediatric practice, there is clearly a need for further studies in this field. Across the studies, there was agreement that oral health screening should be a role for pediatricians, however, there was wide variation in reported rates of oral health assessment. While current guidelines advocate the first dental visit by 1 year of age [48] a lack of awareness of these guidelines and a lack of appropriate referrals was commonly reported. In a number of the studies reviewed, knowledge of fluoride use was explored. Fluoride varnish application for the prevention of carious lesions is supported by evidence [77], yet lack of confidence and low rates fluoride of use were reported. Robust studies that further explore oral health assessment and utilization of interventions such as fluoride varnish are warranted.

The current and potential practice of pediatricians was a particular focus of this review.

There is some evidence of risk-based referral in some of the studies. This may be appropriate assuming risk status of children is being accurately assessed, however, we were unable to determine this from the study reports. The presence of established irreversible disease (i.e. cavitation) was likely the trigger in many cases for referral rather than an actual risk assessment. Although there was limited information reported in these studies, the findings of Long et al. [30] showed that pediatricians were not referring children rated at high risk if they were not yet showing obvious clinical disease. Existing (clinically visible) disease is the best predictor of future disease [78], but it should not be used as a risk indicator exclusively [79]. Primary prevention of disease cannot be achieved this way and more sensitive and early risk assessment and intervention is required [5, 80].

In studies that investigated whether pediatricians gave oral health advice to parents, most respondents indicated they did. Available time during routine consultations was the most frequently reported barrier. Regardless of setting or funding mechanisms, health professionals are faced with challenges in allocating resources (including time) whilst maximizing health outcomes [81]. The value of time spent on oral health, as opposed to more routine and familiar activities may not be appreciated if evidence-based interventions (and the magnitude of their impact) are poorly understood or if remuneration for oral health is low or non-existent. If oral health is to be incorporated into pediatricians’ care, careful thought should be given to how clinicians will be encouraged and supported to do so.

## Conclusion

Pediatricians have an important role in children’s oral health. However, there have been no reviews that have brought together evidence on the knowledge and practice of pediatricians regarding children’s oral health. This review addresses this gap. The findings demonstrate that pediatricians have limited knowledge and understanding in critical areas, including; initial clinical signs of dental caries, recommended age for first dental visit, the transmission of bacteria from mother to child in the etiology of dental caries, and recommended use of fluorides. Barriers to oral health practice for pediatricians include inadequate education and training, time constraints in practice, lack of referral pathways, and cost implications that are often compounded by complicated medical/dental insurance schemes. Addressing gaps in education and training and action on other barriers must be a priority. There is a need for the development of a well-designed validated tool to assess knowledge and practice. This scoping review provides a useful starting point to guide future research and areas for focused, systematic reviews.

## Supplementary information

**Additional file 1.**

## Data Availability

All data generated or analyzed during this study are included in this published article [and its supplementary information files].

## Abbreviations

AAP: American Academy of Pediatrics
WHO: World Health Organization
UN: United Nations
